# Association between secondhand smoke exposure and incidence of metabolic syndrome: analyses of Korean Genome and Epidemiology Study (KoGES) data

**DOI:** 10.4178/epih.e2025041

**Published:** 2025-07-29

**Authors:** Seungmi Choi, Sanghyuk Bae

**Affiliations:** 1 Graduate School of Public Health and Healthcare Management, The Catholic University of Korea, Seoul, Korea; 2 Department of Preventive Medicine, College of Medicine, The Catholic University of Korea, Seoul, Korea

**Keywords:** Tobacco smoke pollution, Metabolic syndrome, Cohort studies

## Abstract

**OBJECTIVES:**

Secondhand smoke exposure remains a major public health concern and is linked to various chronic diseases, including metabolic syndrome (MetS). Although smoking rates have declined, exposure to secondhand smoke remains common and poses significant health risks to non-smokers. This study investigated the association between secondhand smoke exposure and the incidence of MetS using longitudinal data from a community-based cohort in Korea.

**METHODS:**

We utilized data from the Korean Genome and Epidemiology Study, collected biennially between 2001 and 2020. Of 10,030 adults aged 40-69 years, a total of 3,042 never-smokers without pre-existing MetS were included. Secondhand smoke exposure was assessed through self-reported questionnaires. Cox proportional hazards models were employed to estimate hazard ratios (HRs) and 95% confidence intervals (CIs), adjusting for potential confounders.

**RESULTS:**

During follow-up, 638 participants in the secondhand smoke exposure group developed MetS. Those exposed to secondhand smoke had a significantly higher risk of developing MetS compared to the non-exposed group (HR, 1.15; 95% CI, 1.02 to 1.27). Among MetS components, secondhand smoke exposure was significantly associated with increased risks of hypertension (HR, 1.13; 95% CI, 1.00 to 1.28) and hyperglycemia (HR, 1.19; 95% CI, 1.03 to 1.37). Although home exposure was not significantly associated with MetS risk, workplace exposure to secondhand smoke demonstrated a dose-response relationship according to exposure frequency and duration.

**CONCLUSIONS:**

Our findings suggest that secondhand smoke exposure may increase the risk of developing MetS. These results underscore the importance of strengthening regulations on secondhand smoke in public places and raising social awareness of its detrimental effects on non-smokers.

## GRAPHICAL ABSTRACT

[Fig f2-epih-47-e2025041]

## Key Message

In a community-based Korean cohort of 3,042 never-smokers aged 40-69 years, secondhand smoke exposure was associated with a higher risk of developing metabolic syndrome. Risks were particularly elevated for hypertension and hyperglycemia, and workplace exposure showed a dose-response relationship. These findings highlight the need for stronger regulations and public awareness to protect non-smokers from secondhand smoke.

## INTRODUCTION

Secondhand smoke refers to the indirect exposure of non-smokers to harmful substances emitted from tobacco smoke while a smoker is actively smoking [1]. Smoking-related diseases and deaths are among the most significant public health threats worldwide. In Korea alone, more than 60,000 deaths in 2020 were attributed to smoking, while globally, approximately 8 million people die from smoking each year, including about 1.2 million deaths caused by secondhand smoke exposure [1,2].

Research on the harmful effects of secondhand smoke began in earnest in the mid-20th century [3]. International organizations, such as the International Agency for Research on Cancer, classify secondhand smoke as a Group 1 carcinogen [4]. Secondhand smoke contains more than 50 carcinogenic substances, with nicotine, tar, carbon monoxide, and formaldehyde being the primary harmful components [5]. Among these, nicotine is known to be associated with hypertension, dyslipidemia, and insulin resistance [6].

Non-smokers can be exposed to secondhand smoke in a range of indoor and outdoor environments, including homes, workplaces, parks, and streets. Such exposure can lead to various health problems, including respiratory diseases, different types of cancer, cardiovascular diseases, and other chronic conditions [7-9]. Despite declining smoking rates, secondhand smoke continues to pose health risks to non-smokers [7,8,10,11]. Furthermore, there is no safe level of exposure to secondhand smoke; even brief exposure can cause immediate harm, and chronic exposure further elevates the risk of lung cancer and other diseases, comparable to the risks from direct smoking [12]. Therefore, continued attention and interventions to reduce secondhand smoke exposure are essential. In response to these health risks, many countries have introduced regulatory policies, such as bans on smoking in public spaces. Since 2011, Korea has implemented such measures through amendments to the National Health Promotion Act, which restrict smoking in public areas [13]. National statistics indicate that, as of 2021, the rates of secondhand smoke exposure indoors at the workplace and at home among Korean adults aged 19 years or older were 9.2% and 3.6%, respectively. Although indoor workplace exposure has steadily declined since 2013, it still exceeds the rate of home exposure [11].

Globally, the prevalence of metabolic syndrome (MetS) is steadily rising [14,15]. In Korea, the prevalence of MetS increased from 21.1% in 2007 to 24.9% in 2021, showing a steady upward trend. Currently, approximately 1 in 4 adults aged 19 and older is affected by MetS, prompting the implementation of early detection and long-term health management programs in Korea [16-18]. According to the National Cholesterol Education Program-Adult Treatment Panel III (NCEP-ATP III) guidelines, established by the National Institutes of Health (NIH, 2001), MetS is diagnosed when 3 or more of the following conditions are present: hypertension, hyperglycemia, hypertriglyceridemia, low high-density lipoprotein (HDL) cholesterol, and/or abdominal obesity [19]. These risk factors often occur together and are closely related to insulin resistance; thus, MetS was previously termed insulin resistance syndrome [20]. MetS increases the risk of developing cardiovascular disease and diabetes [21].

Previous studies, both in Korea and internationally, have reported that exposure to secondhand smoke increases the risk of MetS [22-26]. Moreover, secondhand smoke exposure is associated with the individual components of MetS. For instance, a Korean cohort study found that individuals exposed to secondhand smoke had a 1.41-fold higher risk of developing diabetes than those not exposed [27]. Additional studies have linked secondhand smoke exposure to health issues related to MetS components, including abdominal obesity, hypertension, hypertriglyceridemia, and low HDL cholesterol [28-30]. These findings indicate that secondhand smoke not only elevates the risk of diseases meeting the MetS diagnostic criteria but may also significantly impact overall metabolic health, extending beyond respiratory or cardiovascular effects. Although there are studies examining the relationship between secondhand smoke exposure and MetS, most have relied on hospital-based health examination cohorts of limited duration or on cross-sectional designs [22,23,25]. Such designs are limited in their ability to clarify the causal relationship between secondhand smoke exposure and the incidence of MetS. Because MetS does not develop over a short time frame, longitudinal studies following the same population over time are required to observe disease progression and related changes. Therefore, this study aimed to investigate the association between secondhand smoke exposure and MetS among Korean adults using community-based cohort data from the Korean Genome and Epidemiology Study (KoGES), which tracks a general population over multiple years to examine the effects of secondhand smoke exposure on chronic disease incidence.

## MATERIALS AND METHODS

### Study participants

The KoGES, conducted by the NIH under the Korea Disease Control and Prevention Agency, began recruiting participants from the general population in 2001 and has since established a large-scale cohort of approximately 235,000 individuals. This study utilized data from the community-based cohort of KoGES. The cohort includes residents aged 40-69 in Ansan and Anseong, Gyeonggi Province, and examines lifestyle, dietary, and environmental factors affecting chronic disease incidence [31]. Baseline survey for the KoGES community-based cohort was conducted from 2001 to 2002 with 10,030 participants, followed by biennial follow-up assessments. This study used 20 years of data, from the baseline survey (2001-2002) to the ninth follow-up (2019-2020).

**Figure 1.**
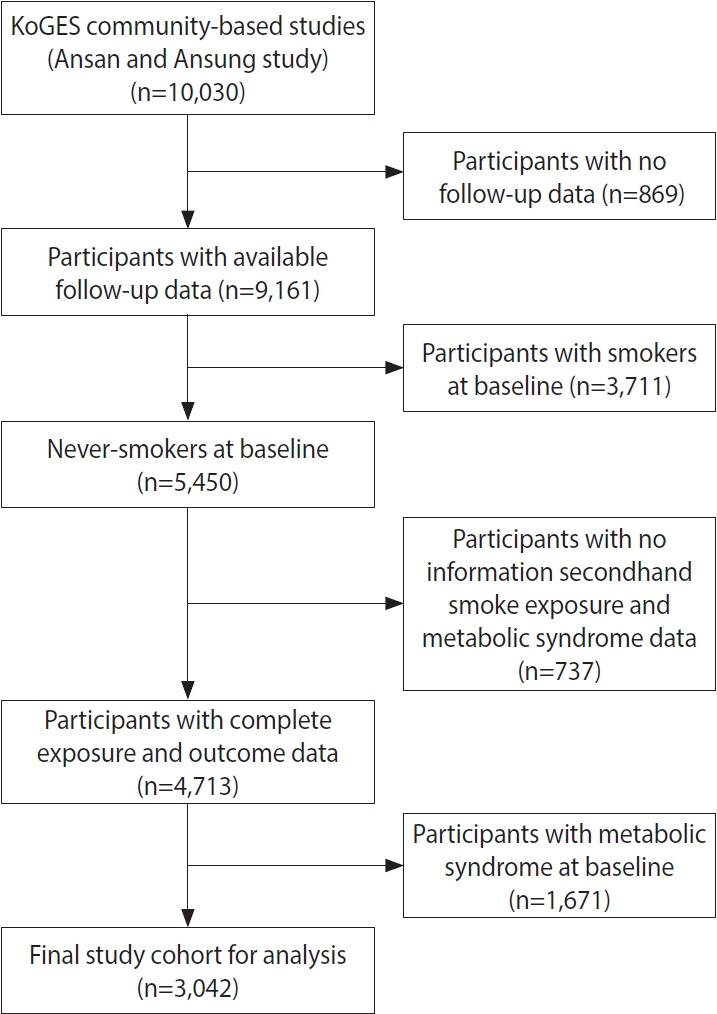
Flowchart showing the selection criteria for study population. KoGES, Korean Genome and Epidemiology Study.

Of the 10,030 participants enrolled in the Ansan and Anseong cohorts at baseline (2001-2002), we excluded those who (1) did not participate in any follow-up assessment after the baseline survey (n=869), (2) were current or former smokers at baseline (n=3,711), (3) lacked information on secondhand smoke exposure and MetS diagnosis (n=737), or (4) had MetS at baseline (n=1,671). No participants started smoking during the follow-up period. Ultimately, 3,042 participants were included in the final analysis (Figure 1). Among these, 1,525 individuals (50.1%) developed MetS during the follow-up period and were censored at the time of event occurrence. A total of 916 participants (30.1%) remained free of MetS and completed follow-up through the ninth survey. The remaining 601 participants (19.8%) were either lost to follow-up or did not participate in the ninth follow-up survey and were censored at their last observed visit.

### Definition of smoking and secondhand smoke exposure

Secondhand smoke exposure status was determined using self-reported survey responses. Smoking status at baseline (2001-2002) was identified through the question, “Do you currently smoke cigarettes?” Participants who answered “never smoked” were classified as non-smokers. Among these non-smokers, those who answered “no” to the question, “Are you currently exposed to secondhand smoke?” were categorized as the non-secondhand smoke exposure group, while those who answered “yes” were classified as the secondhand smoke exposure group. Frequency of secondhand smoke exposure at home and in the workplace was categorized as “none,” “less than 3 days per week,” “3 or more days per week,” and “daily.” Exposure duration was classified as “none,” “less than 1 hour,” and “1 hour or more.” For total secondhand smoke exposure duration, categories were defined as “none,” “less than 5 years,” “5 years to 10 years,” and “10 years or more” for further analysis.

### Definition of metabolic syndrome

MetS was defined according to the NCEP-ATP III guidelines [19], based on anthropometric measurements, body composition, and clinical test results collected biennially from the first follow-up (2003-2004) through the ninth follow-up (2019-2020). The diagnosis of MetS required meeting 3 or more of the following criteria: abdominal obesity (waist circumference ≥90 cm in men and ≥80 cm in women), elevated blood pressure (systolic ≥130 mmHg, diastolic ≥85 mmHg, or use of antihypertensive medication), high fasting glucose (≥100 mg/dL or use of glucose-lowering medication), elevated triglycerides (≥150 mg/dL), and reduced HDL cholesterol (<40 mg/dL in men and <50 mg/dL in women).

### Statistical analysis

To compare general characteristics of participants by secondhand smoke exposure status, categorical variables were analyzed using the chi-square test, with results reported as frequencies and percentages. Continuous variables were assessed using the Wilcoxon rank sum test, with results presented as medians and ranges (minimum-maximum). The incidence of MetS by secondhand smoke exposure status was calculated as the number of cases per 1,000 person-years. Associations between secondhand smoke exposure and the incidence of overall MetS and its components—abdominal obesity, hypertension, hyperglycemia, hypertriglyceridemia, and low HDL cholesterol—were evaluated using Cox proportional hazards models. The timing of event occurrence was estimated using the midpoint imputation method; specifically, for participants who developed MetS, the event time was defined as the midpoint between the last follow-up where MetS was absent and the first follow-up where MetS was diagnosed. For the analysis of individual MetS components, participants with the respective condition at baseline were excluded from the total study population (n=3,042) to ensure inclusion of only those truly at risk for each component. To account for uncertainty in the exact timing of MetS onset, an additional analysis using an interval-censored survival model was conducted. Furthermore, to account for potential changes in secondhand smoke exposure during the long follow-up, a sensitivity analysis was performed based on exposure status at the midpoint of follow-up (4th follow-up survey, 2009-2010), to enhance the reliability of study findings. Dose-response analyses were also performed to assess the relationship between the frequency and duration of secondhand smoke exposure and MetS incidence, and subgroup analyses were conducted by gender. Covariate adjustment was performed using 3 models: the unadjusted model; model 1, adjusted for demographic characteristics (age, gender, household size, occupation type, education level, and income); and model 2, which further adjusted for lifestyle factors (alcohol consumption, physical activity, and body mass index [BMI]) in addition to variables included in model 1. The proportional hazards assumption for secondhand smoke exposure and other covariates was verified using the Supremum test. All statistical analyses were conducted using SAS version 9.4 (SAS Institute Inc., Cary, NC, USA), with statistical significance set at a 2-sided p-value of <0.05.

### Ethics statement

The study received approval from the Institutional Review Board of The Catholic University of Korea (IRB No. MC23EASI0085).

## RESULTS

Baseline characteristics of the study population according to secondhand smoke exposure are presented in Table 1. Among the total 3,042 study participants, 1,253 individuals (41.2%) were exposed to secondhand smoke. The study population comprised 559 men (18.4%) and 2,483 women (81.6%). Statistically significant differences were found in education level and drinking status according to secondhand smoke exposure. Participants in the secondhand smoke exposure group were younger than those in the non-exposure group. Additionally, systolic blood pressure and total cholesterol levels were lower in the secondhand smoke exposure group compared to the non-exposure group. A total of 1,789 participants were classified as the secondhand smoke non-exposure group, among whom 887 individuals developed MetS. The secondhand smoke exposure group consisted of 1,253 participants, with 638 individuals developing MetS. The total observation period for this cohort was 26,598 person-years, with a mean follow-up duration of 8.75 years. Regarding the incidence of Mets according to secondhand smoke exposure status, the incidence rate of MetS was 55.83 cases per 1,000 person-years in the secondhand smoke non-exposure group and 59.57 cases per 1,000 person-years in the secondhand smoke exposure group. The difference in incidence rates between the 2 groups was 3.74 cases per 1,000 person-years, with a slightly higher incidence of MetS observed in the secondhand smoke exposure group. Baseline characteristics of the study population and excluded individuals are shown in Supplementary Material 1.

Table 2 presents the results of the Cox proportional hazards model analyses assessing the associations between baseline secondhand smoke exposure and the incidence of MetS and its components. Compared to the non-exposure group, the secondhand smoke exposure group had a significantly higher risk of developing MetS, even after adjusting for covariates (hazard ratio [HR], 1.15; 95% CI, 1.02 to 1.27). Among the components of MetS, baseline secondhand smoke exposure was significantly associated with a higher risk of hypertension (HR, 1.13; 95% CI, 1.00 to 1.28) and hyperglycemia (HR, 1.19; 95% CI, 1.03 to 1.37) compared to the non-exposure group. Similar results were obtained in sensitivity analyses assessing the association between secondhand smoke exposure and the incidence of MetS using secondhand smoke exposure status at the midpoint of the cohort study (Supplementary Material 2). For women, no significant association was observed between secondhand smoke exposure and the incidence of MetS or its components. For men, although secondhand smoke exposure was not significantly associated with the incidence of MetS, it was associated with an increased risk of hyperglycemia (HR, 1.58; 95% CI, 1.13 to 2.22; Supplementary Material 3).

The dose-response relationship between baseline secondhand smoke exposure frequency and duration at home and in the workplace and the incidence of MetS is shown in Table 3. Daily secondhand smoke exposure at home was associated with an increased risk of MetS compared to the non-exposure group (HR, 1.15; 95% CI, 1.01 to 1.31). However, a consistent trend of increasing risk with higher exposure frequency and longer exposure duration at home was not observed. Participants exposed to secondhand smoke for more than 1 hour at the workplace had a higher risk of developing MetS compared to the non-exposure group (HR, 1.46; 95% CI, 1.17 to 1.82). A dose-response relationship was observed, with the risk of MetS increasing as both the frequency and duration of secondhand smoke exposure at the workplace increased (p for trend=0.024 and <0.001, respectively).

## DISCUSSION

We analyzed the association between secondhand smoke exposure and the incidence of MetS in Korean adults using longitudinal data from a community-based cohort. Analysis based on baseline secondhand smoke exposure status revealed a significant association between secondhand smoke exposure and the incidence of MetS. Furthermore, a sensitivity analysis based on secondhand smoke exposure status at the midpoint of the study (fourth follow-up survey, 2009-2010) showed similar results, consistent with previous studies demonstrating a higher risk of MetS among adults exposed to secondhand smoke. For example, a cohort study of Korean adults in their 30s who underwent health checkups found that continuous secondhand smoke exposure increased the risk of MetS by 1.19-fold [22]. Similarly, international research has reported an elevated incidence of MetS among individuals with secondhand smoke exposure for 5-7 days per week [23]. To account for uncertainty in the exact timing of MetS onset, we also conducted an interval-censored survival analysis. Although the results were not statistically significant, the direction and magnitude of the association were consistent with the main findings, supporting the robustness of our conclusions (Supplementary Material 4). In this study, we adjusted for a wide range of demographic and lifestyle covariates and also calculated the E-value to assess the potential impact of unmeasured confounding. The E-value for the observed HR of 1.15 was 1.57. To further evaluate the potential influence of unmeasured confounding, we reviewed prior studies examining the associations between relevant factors and MetS. For instance, a study of dietary patterns and MetS among Korean women found an inverse association only for the “prudent” dietary pattern, while the “western” pattern was not significantly associated with MetS [32]. Additionally, a meta-analysis investigating the association between depressive symptoms and MetS reported a pooled relative risk of 1.29 (95% CI, 1.12 to 1.48), with significant associations observed in Western countries but not in Asian populations [33]. These estimates fall below the E-value threshold of 1.57. Therefore, although unmeasured confounders may still exist, it is unlikely that any single factor would be strong enough to fully account for the observed association.

Our study also found that secondhand smoke exposure significantly increased the risk of hypertension and hyperglycemia, 2 components of MetS. These findings are consistent with previous research demonstrating an association between secondhand smoke exposure, as assessed via urinary cotinine levels, and an increased risk of hypertension [28]. Additionally, a domestic cohort study reported that secondhand smoke exposure increased the risk of type 2 diabetes by 1.41-fold compared to non-exposure [27]. However, our study did not find statistically significant associations between secondhand smoke exposure and other MetS components, including abdominal obesity, hypertriglyceridemia, and low HDL-cholesterol. Similarly, a previous Korean study that evaluated secondhand smoke exposure using both self-reports and urinary cotinine levels found no significant associations between secondhand smoke exposure and hypertriglyceridemia or low HDL cholesterol [34]. These findings are in line with those of the current study. Nonetheless, other international studies have reported significant associations. For example, a study on Chinese adults found that secondhand smoke exposure increased the risk of abdominal obesity (odds ratio [OR], 2.7; 95% CI, 1.6 to 4.5), hypertriglyceridemia (OR, 2.1; 95% CI, 1.1 to 3.9), and low HDL cholesterol (OR, 1.9; 95% CI, 1.1 to 3.1) [23]. Another study involving never-smoking cancer survivors in Korea reported a significant association between secondhand smoke exposure and hypertriglyceridemia (OR, 1.63; 95% CI, 1.07 to 2.48), but not other MetS components [35]. Thus, the association between secondhand smoke exposure and the components of MetS remains inconsistent across studies.

Several possible explanations may account for these inconsistent findings. First, publication bias may be present, as studies reporting non-significant associations are less likely to be published. Second, differences in study populations may have influenced the results. For example, the study of Chinese adults included current smokers and adjusted for smoking status as a confounder, while in the present study, we excluded smokers to assess the independent effects of secondhand smoke exposure among non-smokers. Third, most previous studies have been cross-sectional, limiting their ability to establish causal relationships. In contrast, our study utilized long-term cohort data, providing stronger evidence for the association between secondhand smoke exposure and MetS components.

In the present study, dose-response analysis revealed that prolonged secondhand smoke exposure in the workplace significantly increased the risk of MetS. Participants exposed to secondhand smoke for more than 1 hour daily at work had a higher risk of MetS than those without exposure, and the risk increased with both higher frequency and longer exposure duration. However, no significant associations were observed for secondhand smoke exposure at home or for cumulative exposure duration. These findings differ from some prior studies that reported a significant increase in MetS risk with longer cumulative exposure [22,25]. However, those previous studies were cross-sectional or based on short-term health examination cohorts, limiting their ability to determine causal relationships. Additionally, the total duration of secondhand smoke exposure in our study was assessed via self-report, which may not have fully captured actual exposure. This result may also be explained by the limited implementation of indoor smoking bans in Korea, where restrictions have applied only to workplaces larger than 1,000 m² since 2012 [11,13]. As a result, secondhand smoke exposure in workplaces may remain high, leading to prolonged exposure in confined and poorly ventilated spaces. These findings suggest that workplace secondhand smoke exposure may significantly increase the risk of developing MetS.

The exact mechanisms underlying the increased risk of MetS with secondhand smoke exposure remain unclear. However, non-smokers exposed to secondhand smoke can absorb harmful chemicals similar to those absorbed by active smokers. According to the National Toxicology Program in the United States, secondhand smoke contains more than 50 carcinogens and 250 toxic chemicals [36]. Prolonged exposure to secondhand smoke generates reactive oxygen species, increasing oxidative stress and leading to chronic inflammation, which is closely linked to MetS and diabetes [37,38]. Additionally, nicotine-induced chronic inflammation and stimulation of the sympathetic nervous system may lead to insulin resistance, resulting in elevated blood glucose and triglyceride levels and reduced HDL cholesterol levels [39,40]. Nicotine also alters cyclic adenosine monophosphate signaling, potentially impairing vascular function and contributing to metabolic and vascular complications [41]. Furthermore, secondhand smoke-induced cortisol release has been associated with increased abdominal obesity [42]. These mechanisms collectively contribute to the development and progression of MetS in individuals exposed to secondhand smoke.

Our study has several strengths. First, we utilized community-based cohort data from KoGES, allowing for the analysis of secondhand smoke exposure and MetS incidence in a general population rather than a specific subgroup. Second, the study design excluded active smokers at baseline, providing a more precise evaluation of the independent effects of secondhand smoke exposure among a population of consistent never-smokers throughout the follow-up period. While most previous studies were cross-sectional or based on short-term health screening cohorts, this study provides more robust evidence regarding the metabolic health effects of secondhand smoke by utilizing a large, community-based cohort of never-smokers, distinguishing secondhand smoke exposure at home and in the workplace, and evaluating dose-response relationships according to exposure intensity. Additionally, by focusing on MetS as a composite outcome rather than its individual components, this study offers a more comprehensive understanding of the cumulative metabolic risk associated with secondhand smoke exposure. This approach reflects the clinical relevance of MetS as a cluster of co-occurring metabolic abnormalities and strengthens the interpretation of long-term health impacts.

However, several limitations must be acknowledged. First, both secondhand smoke exposure and smoking status were assessed through self-reported questionnaires, which may have introduced misclassification due to under-reporting or over-reporting, particularly among socially sensitive subgroups such as women. Future research should incorporate objective validation tools, such as urinary or serum cotinine levels, to improve the accuracy of secondhand smoke exposure assessment. Second, participant attrition occurred during the 20-year follow-up period, with a retention rate of 58%. Despite this limitation, the use of Korea’s longest-running cohort data provides valuable insights. Third, since the onset of MetS was determined based on scheduled follow-up assessments rather than exact dates, the precise timing of disease occurrence could not be established. To address this limitation, we applied a conservative approach using the midpoint imputation method to estimate the timing of MetS onset. Fourth, our analysis relied on baseline secondhand smoke exposure data, which limited our ability to account for changes in exposure over time. To address this, we conducted a sensitivity analysis using secondhand smoke exposure status at the intermediate time point (the 4th follow-up), which helped to enhance the reliability of our findings. Finally, despite adjusting for multiple covariates, the possibility of residual confounding due to unmeasured or imprecisely measured factors, such as dietary intake, psychological stress, environmental pollutants, or genetic predisposition, cannot be excluded. Future research should incorporate these factors to obtain a more comprehensive understanding of the association between secondhand smoke exposure and MetS.

In conclusion, we observed a significant association between secondhand smoke exposure and increased MetS risk based on analyses of the longest-running cohort study data in Korea (ongoing for over 20 years). Among the MetS components, secondhand smoke exposure was most strongly associated with increased risks of hypertension and hyperglycemia. These findings highlight the harmful effects of secondhand smoke exposure on non-smokers and underscore the need for stricter secondhand smoke regulations in public spaces and increased public awareness. Our results provide a foundation for developing evidence-based public health policies aimed at mitigating the impacts of secondhand smoke exposure.

## Figures and Tables

**Figure f2-epih-47-e2025041:**
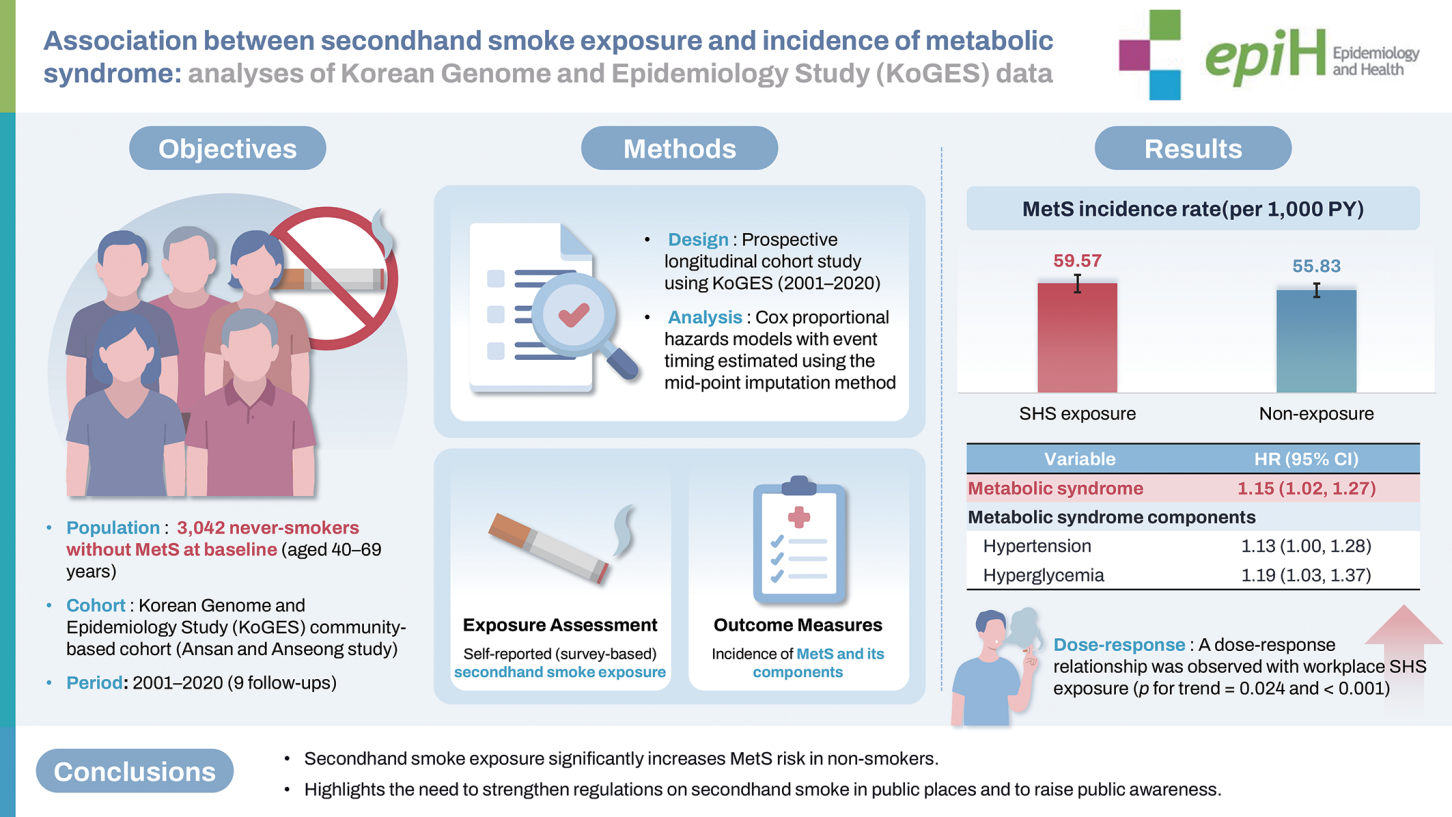


**Table 1.** Baseline characteristics of the study population

Characteristics	SHS exposure		p-value^1^
	No (n=1,789)	Yes (n=1,253)	
Gender			<0.001
Men	360 (64.4)	199 (35.6)	
Women	1,429 (57.5)	1,054 (42.4)	
Resident area			0.451
Ansung (rural)	701 (38.2)	508 (40.5)	
Ansan (urban)	1,088 (60.8)	745 (59.5)	
Education			<0.001
≤Elementary school	554 (31.0)	338 (27.1)	
≤High school	1,004 (56.25)	790 (63.3)	
Over college	227 (12.7)	120 (9.6)	
Income (Korean won)			0.059
<1,000,000	539 (30.6)	329 (26.7)	
1,000,000-1,990,000	549 (31.1)	387 (31.4)	
2,000,000-3,999,000	548 (31.1)	408 (33.1)	
≥4,000,000	127 (7.2)	110 (8.9)	
Regular exercise			0.896
No	847 (48.1)	592 (48.4)	
Yes	913 (51.9)	632 (51.6)	
Drinking status			<0.001
No	1,185 (66.3)	689 (55.2)	
Past	52 (2.9)	44 (3.5)	
Current	549 (30.7)	514 (41.2)	
Body mass index (kg/m²)			0.068
<25	1,226 (68.9)	819 (65.7)	
≥25	554 (31.1)	427 (34.3)	
Age (yr)	49.0 (40.0-69.0)	46.0 (40.0-69.0)	<0.001
Waist circumference (cm)	77.0 (56.0-110.5)	77.0 (56.0-112.0)	0.465
Systolic blood pressure (mmHg)	112.0 (70.0-200.0)	110.0 (72.0-210.0)	0.006
Diastolic blood pressure (mmHg)	74.0 (40.0-120.0)	74.0 (40.0-122.0)	0.300
Triglycerides (mg/dL)	107.0 (36.0-885.0)	105.0 (40.0-864.0)	0.267
HDL-C (mg/dL)	48.0 (25.0-96.0)	47.0 (23.0-95.0)	0.519
Glucose (mg/dL)	80.0 (62.0-276.0)	81.0 (45.0-241.0)	0.165
Creatinine (mg/dL)	0.7 (0.5-1.4)	0.7 (0.5-2.0)	0.050
Total cholesterol (mg/dL)	186.0 (44.0-300.0)	181.0 (80.0-369.0)	<0.001
CRP (mg/dL)	0.11 (0.01-7.59)	0.12 (0.01-9.23)	0.102
Incident cases	887 (58.16)	638 (41.84)	
Person-years	15,888.7	10,709.6	
Incidence rate (95% CI)^2^	55.83 (52.21, 59.62)	59.57 (55.04, 64.38)	

Values are presented as number (%) or median (minimum-maximum). SHS, secondhand smoke; HDL-C, high-density lipoprotein cholesterol; CRP, C-reactive protein; CI, confidence interval.

1 Calculated using the chi-square test for categorical variables and the Wilcoxon rank sum test for continuous variables.

2 Per 1,000 person-years.

**Table 2.** Association of secondhand smoke exposure^1^ at baseline with metabolic syndrome in 2001-2020 KoGES participants (n=3,042)

Variables	Crude	Model 1^2^	Model 2^3^
Metabolic syndrome	1.29 (1.16, 1.43)	1.17 (1.05, 1.30)	1.15 (1.02, 1.27)
Metabolic syndrome components			
Abdominal obesity (n=2,151)	1.32 (1.17, 1.48)	1.12 (0.99, 1.26)	1.09 (0.96, 1.23)
Hypertension (n=2,368)	1.22 (1.09, 1.37)	1.16 (1.02, 1.30)	1.13 (1.00, 1.28)
Hyperglycemia (n=2,946)	1.26 (1.10, 1.44)	1.23 (1.07, 1.41)	1.19 (1.03, 1.37)
Hypertriglyceridemia (n=2,588)	1.10 (0.96, 1.24)	0.96 (0.84, 1.09)	0.94 (0.82, 1.06)
Low HDL-C (n=1,533)	1.09 (0.95, 1.25)	0.87 (0.75, 1.00)	0.86 (0.74, 1.00)

Values are presented as hazard ratio (95% confidence interval). KoGES, Korean Genome and Epidemiology Study; HDL-C, high-density lipoprotein cholesterol.

1 The reference group was the no secondhand smoke exposure group.

2 Model 1: adjusted for age, gender, household size, occupation type, education level and income.

3 Model 2: adjusted for model 1+alcohol consumption, regular exercise, and body mass index.

**Table 3.** Dose-response relationship between SHS exposure at baseline and metabolic syndrome by exposure place in 2001-2020 KoGES participants (n=3,042)

Variables	Crude	Model 1^1^	Model 2^2^	p for trend
Frequency of SHS exposure (day/wk)				
Home				0.861
No exposure	1.00 (reference)	1.00 (reference)	1.00 (reference)	
<3	1.45 (1.18, 1.78)	1.26 (1.01, 1.55)	1.13 (0.90,1.39)	
3-6	1.41 (0.98, 2.01)	1.15 (0.80,1.64)	1.16 (0.80,1.67)	
Every day	1.26 (1.12, 1.42)	1.15 (1.01,1.31)	1.15 (1.01,1.31)	
Work				0.024
No exposure	1.00 (reference)	1.00 (reference)	1.00 (reference)	
<3	1.25 (0.93, 1.68)	1.42 (1.04, 1.92)	1.41 (1.04, 1.92)	
3-6	0.70 (0.38, 1.26)	0.61 (0.32, 1.13)	0.68 (0.36, 1.27)	
Every day	1.37 (1.22, 1.65)	1.30 (1.06, 1.60)	1.18 (0.95, 1.45)	
Duration of daily SHS exposure (hr)				
Home				0.186
No exposure	1.00 (reference)	1.00 (reference)	1.00 (reference)	
<1	1.27 (1.14, 1.45)	1.11 (0.96, 1.28)	1.10 (0.95, 1.26)	
≥1	1.20 (1.02, 1.41)	1.17 (1.00, 1.38)	1.17 (0.99, 1.37)	
Work				<0.001
No exposure	1.00 (reference)	1.00 (reference)	1.00 (reference)	
<1	0.94 (0.64, 1.10)	0.84 (0.63, 1.10)	0.80 (0.60, 1.05)	
≥1	1.67 (1.36, 2.03)	1.61 (1.29, 1.99)	1.46 (1.17, 1.82)	
Duration of SHS exposure (yr)				0.841
No exposure	1.00 (reference)	1.00 (reference)	1.00 (reference)	
<5	1.73 (1.08, 2.76)	1.67 (1.04, 2.69)	1.41 (0.87, 2.27)	
5≤ exposure <10	2.62 (1.24, 5.52)	2.65 (1.24, 5.64)	2.67 (1.25, 5.71)	
≥10	1.52 (1.26, 1.83)	1.34 (1.09, 1.63)	1.23 (1.00, 1.49)	

Values are presented as hazard ratio (95% confidence interval). SHS, secondhand smoke; KoGES, Korean Genome and Epidemiology Study.

1 Model 1: adjusted for age, gender, household size, occupation type, education level and income.

2 Model 2: adjusted for model 1+alcohol consumption, regular exercise, and body mass index.

## Supplementary materials

Supplementary Material 1. Baseline Characteristics of Included and Excluded Participants(N=10,030)

Supplementary Material 2. Sensitivity analysis of the association between secondhand smoke exposure at follow-up (2009-2010) and metabolic syndrome (N=2,271)

Supplementary Material 3. Association between secondhand smoke exposure at baseline and metabolic syndrome in man and woman

Supplementary Material 4. Interval-Censored Survival Analysis of Secondhand Smoke Exposure and Metabolic Syndrome Incidence(N=3,042)
