# Supplementary material for: Association between secondhand smoke exposure and incidence of metabolic syndrome: analyses of Korean Genome and Epidemiology Study (KoGES) data
Source: Epidemiol Health. 2025 Jul 29;47:e2025041. doi: 10.4178/epih.e2025041 (PMC12869143; doi:10.4178/epih.e2025041)
Supplement: Supplementary Material 1. — Baseline Characteristics of Included and Excluded Participants(N=10,030) [file epih-47-e2025041-Supplementary-1.docx]

Supplementry Material 1. Baseline Characteristics of Included and Excluded Participants(N=10,030)

| Variables | Man(N=4758) | | P value* | Woman(N=5272) | | P value* |
| --- | --- | --- | --- | --- | --- | --- |
|  | Included Participants (n=559) | Excluded Participants (n=4199) |  | Included Participants (n=2483) | Excluded Participants (n=2789) |  |
| Resident area |  |  | <0.001 |  |  | <0.001 |
| Ansung(rural) | 200 (35.78) | 3809 (54.51) |  | 1009 (40.64) | 1769 (63.43) |  |
| Ansan(urban) | 359 (64.22) | 3179 (45.49) |  | 1474 (59.36) | 1020 (36.43) |  |
| Education |  |  | 0.002 |  |  | <0.001 |
| ≤Elementary school | 102 (18.28) | 870 (20.85) |  | 790 (31.92) | 1593 (58.10) |  |
| ≤High school | 308 (55.20) | 2458 (58.92) |  | 1486 (60.04) | 1035 (37.75) |  |
| Over college | 148 (26.52) | 844 (20.23) |  | 199 (8.04) | 114 (4.16) |  |
| Income, KRW |  |  | 0.022 |  |  | <0.001 |
| <1,000,000 | 127 (22.97) | 1213 (29.22) |  | 741 (30.32) | 1440 (53.35) |  |
| 1,000,000~1,990,000 | 179 (32.37) | 1265 (30.47) |  | 757 (30.97) | 691 (25.60) |  |
| 2,000,000~3,999,000 | 189 (34.18) | 1291 (31.10) |  | 767 (31.38) | 458 (16.97) |  |
| ≥4,000,000 | 58 (10.49) | 382 (9.20) |  | 179 (7.32) | 110 (4.08) |  |
| Secondhand smoke |  |  | 0.129 |  |  | 0.883 |
| No | 360 (64.40) | 2226 (61.05) |  | 1429 (57.55) | 1437 (57.76) |  |
| Yes | 199 (35.60) | 1420 (38.95) |  | 1054 (42.45) | 1051 (42.24) |  |
| Regular exercise |  |  | 0.095 |  |  | 0.003 |
| No | 245 (44.87) | 1987 (48.67) |  | 1194 (48.97) | 1414 (53.04) |  |
| Yes | 301 (55.13) | 2096 (51.33) |  | 1244 (51.03) | 1252 (49.96) |  |
| Drinking status |  |  | <0.001 |  |  | <0.001 |
| No | 188 (33.63) | 690 (16.53) |  | 1686 (68.15) | 2032 (74.35) |  |
| Past | 40 (7.16) | 451 (10.81) |  | 56 (2.26) | 105 (3.84) |  |
| Current | 331 (59.21) | 3032 (72.66) |  | 732 (29.59) | 596 (21.81) |  |
| BMI |  |  | <0.001 |  |  | <0.001 |
| BMI < 25g/m² | 379 (68.54) | 2478 (59.68) |  | 2045 (67.58) | 3607 (52.10) |  |
| BMI ≥ 25g/m² | 174 (31.46) | 1674 (40.32) |  | 981 (32.42) | 3316 (47.90) |  |
| Aag (years) | 49.0 (40.0 - 69.0) | 50.0 (40.0 -69.0) | 0.058 | 47.0 (40.0 - 69.0) | 56.0 (40.0 -69.0) | <0.001 |
| Waist circumference (cm) | 82.0 (61.0 - 102.0) | 84.0 (60.0 - 111.5) | <0.001 | 76.0 (56.0 - 112.0) | 85.5 (58.0 - 122.0) | <0.001 |
| Systolic blood pressure (mmHg) | 118.0 (76.0 - 188.0) | 120.0 (70.0 - 230.0) | <0.001 | 110.0 (70.0 - 210.0) | 128.0 (74.0 - 212.0) | <0.001 |
| Diastolic blood pressure (mmHg) | 80.0 (42.0 - 122.0) | 80.0 (40.0 - 138.0) | 0.121 | 74.0 (40.0 - 120.0) | 82.0 (40.0 - 160.0) | <0.001 |
| Triglycerides (mg/dl) | 117.0 (40.0 - 864.0) | 151.0 (32.0 - 1528.0) | <0.001 | 104.0 (36.0 - 885.0) | 160.0 (44.0 - 1557.0) | <0.001 |
| HDL-C (mg/dl) | 44.0 (23.0 - 95.0) | 42.0 (16.0 - 95.0) | <0.001 | 48.0 (24.0 - 96.0) | 41.0 (19.0 - 87.0) | <0.001 |
| Glucose (mg/dl) | 83.0 (63.0 - 241.0) | 85.0(53.0 - 488.0) | <0.001 | 80.0 (45.0 - 276.0) | 82.0 (51.0 - 461.0) | <0.001 |
| Creatinine (mg/dl) | 0.9 (0.6 - 2.0) | 0.9 (0.4 - 13.0) | 0.667 | 0.7 (0.5 - 1.3) | 0.7 (0.5 - 5.4) | 0.612 |
| Total cholesterol (mg/dl) | 189.0 (88.0 - 327.0) | 189.0 (74.0 - 382.0) | 0.538 | 183.0 (44.0 - 369.0) | 193.0 (105.0 - 400.0) | <0.001 |
| CRP (mg/d) | 0.12 (0.01 - 9.2) | 0.15 (0.01 - 15.3) | <0.001 | 0.11 (0.01 - 7.8) | 0.16 (0.01 - 23.8) | <0.001 |
| Data are shown as number (%) or median (min-max).  SHS: Secondhand smoke; KRW: Korean won; BMI: Body mass index; HDL-C: high-density lipoprotein cholesterol; CRP: C-reactive protein.  *Calculated using the chi-square test for categorical variables and the Wilcoxon rank sum test for continuous variables. | | | | | | |
