# Supplementary material for: Association between secondhand smoke exposure and incidence of metabolic syndrome: analyses of Korean Genome and Epidemiology Study (KoGES) data
Source: Epidemiol Health. 2025 Jul 29;47:e2025041. doi: 10.4178/epih.e2025041 (PMC12869143; doi:10.4178/epih.e2025041)
Supplement: Supplementary Material 2. — Sensitivity analysis of the association between secondhand smoke exposure at follow-up (2009-2010) and metabolic syndrome (N=2,271) [file epih-47-e2025041-Supplementary-2.docx]

Supplementry Material 2. Sensitivity analysis of the association between secondhand smoke exposure at follow-up (2009-2010) and metabolic syndrome (N=2,271)

| Variables | Total (n) | Cases (n) | Person-Year | Crude HR (95% CI) | Adjusted HR (95% CI) | | | |
| --- | --- | --- | --- | --- | --- | --- | --- | --- |
|  |  |  |  |  | Model 1* HR (95% CI) | | Model 2† HR (95% CI) | |
| No exposure | 1884 | 1025 | 19,212 | 1 | 1 | | 1 | |
| SHS exposure | 387 | 216 | 13,163 | 1.43 (1.23, 1.65) | 1.17 (1.00, 1.36) | | 1.17 (1.00, 1.36) | |
| HR: Hazard ratio; CI: confidence interval; SHS: Secondhand smoke. | | | | | |  | |  |
| *Model 1: adjusted for age, sex, household size, occupation type, education level and income. | | | | | | | |  |
| †Model 2: adjusted for Model 1 + alcohol consumption, regular exercise and BMI. | | | | | |  | |  |
