# Supplementary material for: Association between secondhand smoke exposure and incidence of metabolic syndrome: analyses of Korean Genome and Epidemiology Study (KoGES) data
Source: Epidemiol Health. 2025 Jul 29;47:e2025041. doi: 10.4178/epih.e2025041 (PMC12869143; doi:10.4178/epih.e2025041)
Supplement: Supplementary Material 3. — Association between secondhand smoke exposure at baseline and metabolic syndrome in man and woman [file epih-47-e2025041-Supplementary-3.docx]

Supplementry Material 3. Association between secondhand smoke exposure at baseline and metabolic syndrome in man and woman

| Variable | Man (N = 559) | | | | | | | | Woman (N = 2483) | | | | | | |
| --- | --- | --- | --- | --- | --- | --- | --- | --- | --- | --- | --- | --- | --- | --- | --- |
|  | Incidence  Cases(n) | Crude HR  (95% CI) | | Model 1^*^ HR  (95% CI) | | Model 2^†^ HR  (95% CI) | | Incidence  Cases(n) | Crude HR  (95% CI) | | Model 1^*^ HR  (95% CI) | | Model 2^†^ HR  (95% CI) | | |
| Metabolic syndrome | 85 | 1.43 (1.09, 1.87) | | 1.14 (0.85, 1.51) | | 1.19 (0.87, 1.61) | | 553 | 1.23 (1.10, 1.37) | | 1.14 (1.01, 1.28) | | 1.12 (0.99, 1.25) | | |
| MetS components |  | |  | |  | |  | |  | | |  | | |  |
| Abdominal obesity | 64 | 1.27 (0.93, 1.72) | | 1.04 (0.75, 1.45) | | 1.05 (0.74, 1.49) | | 414 | 1.27 (1.12, 1.44) | | 1.11 (0.97, 1.27) | | 1.08 (0.95, 1.24) | | |
| Hypertension | 74 | 1.54 (1.15, 2.06) | | 1.21 (0.87, 1.68) | | 1.18 (0.84, 1.64) | | 438 | 1.18 (1.04, 1.34) | | 1.09 (0.95, 1.24) | | 1.07 (0.93, 1.22) | | |
| Hyperglycemia | 74 | 1.70 (1.26, 2.77) | | 1.58 (1.14, 2.18) | | 1.58 (1.13, 2.22) | | 303 | 1.21 (1.04, 1.40) | | 1.16 (0.99, 1.35) | | 1.13 (0.96, 1.32) | | |
| High Triglycerides | 54 | 1.40 (1.00, 1.96) | | 0.92 (0.63, 1.34) | | 0.79 (0.53, 1.17) | | 356 | 1.04 (0.91, 1.19) | | 0.97 (0.84, 1.11) | | 0.94 (0.81, 1.08) | | |
| Low HDL-C | 64 | 1.32 (0.98, 1.79) | | 0.98 (0.69, 1.34) | | 0.97 (0.67, 1.38) | | 258 | 0.99 (0.84, 1.15) | | 0.84 (0.71, 0.98) | | 0.84 (0.71, 0.98) | | |
| The reference group was the no secondhand smoke exposure group. | | | | | | | | | |  | | | |  |  |
| HR: Hazard ratio; CI: confidence interval; MetS: Metabolic syndrome; HDL-C: high-density lipoprotein cholesterol. | | | | | | | | | | | | | |  |  |
| *Model 1: adjusted for age, household size, occupation type, education level and income. | | | | | | | | | | | | | |  |  |
| †Model 2: adjusted for Model 1 + alcohol consumption, regular exercise and BMI. | | | | | | | | | | | | | |  |  |
