# Supplementary material for: Association between secondhand smoke exposure and incidence of metabolic syndrome: analyses of Korean Genome and Epidemiology Study (KoGES) data
Source: Epidemiol Health. 2025 Jul 29;47:e2025041. doi: 10.4178/epih.e2025041 (PMC12869143; doi:10.4178/epih.e2025041)
Supplement: Supplementary Material 4. — Interval-Censored Survival Analysis of Secondhand Smoke Exposure and Metabolic Syndrome Incidence(N=3,042) [file epih-47-e2025041-Supplementary-4.docx]

Supplementry Material 4. Interval-Censored Survival Analysis of Secondhand Smoke Exposure and Metabolic Syndrome Incidence(N=3,042)

| Variables | Crude HR (95% CI) | Adjusted HR (95% CI) | |
| --- | --- | --- | --- |
|  |  | Model 1* HR (95% CI) | Model 2† HR (95% CI) |
| Metabolic syndrome | 1.53 (1.41, 1.66) | 1.29 (0.88, 1.88) | 1.22 (0.82, 1.80) |
| HR: Hazard ratio; CI: confidence interval. | | | |
| *Model 1: adjusted for age, sex, household size, occupation type, education level and income. | | | |
| †Model 2: adjusted for Model 1 + alcohol consumption, regular exercise and BMI. | | | |
